# The Story of the Insane from Year to Year

**Published:** 1897-07-17

**Authors:** 


### THE STORY OF THE INSANE FROM YEAR TO YEAR.

Tenth Series.

### CORNWALL COUNTY ASYLUM AT BODMIN.

The numbers were almost stationary, being 760 at the beginning of the year and 762 at the close of it. There were 92 first admissions, 18 readmissions, 44 recoveries, and 56 deaths. Calculated on the admissions, the recoveries stand at 41·5 per cent. and the deaths at 7·3 on the average number resident. The former is above the county asylum average, and the latter considerably under it. Of the deaths, 13 were caused by cerebral and spinal diseases, 7 by consumption, and 1 by enteric fever, Of the year's admissions, 10 were driven insane by domestic troubles, 11 by religion, 4 by intemperance in drink, and 20 by hereditary influence. Like so many others, the asylum is full, and, indeed, overcrowded. Although this increase in the number of Cornwall patients must have been perfectly well known for many years back, nothing seems to have been done, and it is curious to notice how often boards of management procrastinate over the necessary work of finding accommodation for their insane poor, with the almost invariable result that both time and money are wasted. Dr. Adams says that "Throughout the year the asylum has been terribly overcrowded. The women's wards have on some occasions been full, and admission of patients has been delayed for want of room. Application has been made to the asylums in the South-Western District for accommodation; but all are full and unable to spare us any beds." Even could room be obtained there would be inconvenience to the patients' friends and loss of money to the county to the extent of at least £10 a year for each patient boarded out. Dr. Adams has had a long and honourable service at the Bodmin Asylum, and it is not to be wondered at if he sighs for rest from the anxieties of such a responsible post. Most medical superintendents make the fatal mistake of remaining too long at their work; but there is always a wrench in giving up an habitual and cherished labour, and it is that which keeps so many at the oar. Farming is not a bad business in spite of all that has been said. This asylum cleared £288 last year; but we do not learn from the balance-sheet what prices were charged for the articles supplied to the house. The average weekly cost per head was 10s. 2¼d.

